# Investigation of grafting silane coupling agents on superhydrophobicity of carbonyl iron/SiO_2_ particles for efficient oil/water mixture and emulsion separation

**DOI:** 10.1038/s41598-023-28131-z

**Published:** 2023-01-16

**Authors:** Yahya Rabbani, Hadi Shayesteh, Nima Haghshenas, Mobin Safarzadeh Khosrowshahi

**Affiliations:** 1 School of Chemical Engineering, College of Engineering, University of Tehran (UT), Tehran, Iran; 2 School of Chemical, Petroleum and Gas Engineering, Iran University of Science and Technology (IUST), Tehran, Iran; 3 School of Mechanical Engineering, College of Engineering, University of Tehran (UT), Tehran, Iran; 4 Nanotechnology Department, School of Advanced Technologies, Iran University of Science and Technology (IUST), Tehran, Iran

**Keywords:** Chemical engineering, Environmental chemistry, Environmental chemistry, Surface chemistry

## Abstract

The present study demonstrated the wettability properties of grafting silane coupling agents on carbonyl iron (CI)/SiO_2_ particles for efficient oil/water mixture and emulsion separation. CI particles were first reacted with Tetraethoxysilane (TEOS) to create a magnetic component. Then, CI/SiO_2_ particles were altered by 1H,1H,2H,2H-perfluorodecyltriethoxysilane (FAS) and Hexamethyldisilazane (HDMS) to create magnetic superhydrophobic/superoleophilic, recyclable, and reusable sorbent powders. The water contact angle (WCA) values of the as-prepared particles, CI, CI/SiO_2_, CI/SiO_2_@FAS, and CI/SiO_2_@HMDS, were 5.4° ± 1.3°, 6.4° ± 1.4°, 151.9° ± 2.1°, and 170.1° ± 1.1°, respectively. In addition, the oil contact angles (OCAs) of a variety of oils were found to be equivalent to 0°. Hence, superhydrophobic/superoleophilic particles for kind of different oils were shown sorption capacities of 1.7–3.1 g/g and 2.5–4.3 g/g for CI/SiO_2_@FAS, and CI/SiO_2_@HMDS, respectively. Besides, for 1%w/w hexane/water emulsion separation efficiency higher than 99%, the lowest mass was obtained at 50 and 200 mg for CI/SiO_2_@HDMS and CI/SiO_2_@HDMS, respectively, suggesting a new effective material for separating tiny oil droplets. Also, the reusability and chemical durability of the superhydrophobic samples made them a prime candidate for use in different harsh conditions.

## Introduction

In today’s world, the amount of wastewater produced is increasing dramatically due to the development of various industrious and booming populations globally^1–4^. Industrial wastewater discharge and oil spills in the marine environment not only threaten ecosystems and human health but also destroy a wide range of Earth's natural resources, which motivates researchers to develop proactive, drastic, and solution-focused strategies to mitigate these serious environmental issues^5–7^. So far, numerous materials with various properties have been produced for oil and water separation. Synthesized materials for separation should have the requisite surface qualities such as high surface area, high wettability or superhydrophobicity, good durability, and so on^8–13^.

Wetting and anti-wetting properties of solid surfaces are one of the most common natural phenomena that we see widely in the environment just like dew on plants or the water droplets on some species of insects’ wings that artificial type for the first time was introduced as super-anti wetting property by Ollivier^14^. A Superhydrophobic surface with a high apparent contact angle (> 150°) is commonly used in the form of mesh and porous materials for oil and water separations^15^. These materials suffer from some drawbacks including time-consuming synthesis processes, high cost, and low efficiency, that are considered obstacles to their industrial applications^9,16–19^. Hence, developing simple, scalable, and low-cost fabrication methods is of great importance for the commercial scale of separation projects^9^. The majority of conducted investigations on the hydrophobicity issues have concerned the fabrication methods and processes, the theories behind the unique wettability and non-wettability as well as their applications^14^.

Various methods and strategies have been introduced to fabricate different materials with excellent superhydrophobicity, such as chemical vapor deposition^20^, phase separation^21^, layer-by-layer assembly, electrospinning deposition^22^, colloid assembly^23^, chemical etching^24^, etc.^25,26^. In terms of the mechanism, silanes that do not have hydrolyzable groups, such as Si-CI, Si-OCH_3_, Si-OCH_2_CH_3_, and Si-NH-Si, react with water to create silanols, which are then coupled to hydroxyl groups on the surface of materials. Some of the most important factors that should be considered for the production and modification of superhydrophobic materials include surface roughness and low surface energy of materials^25^. Organic materials with superhydrophobicity typically are in the form of powder or 3D porous sponges to separate water and oil^27^. Also, they can be produced as porous flat films or coated on the meshes^28^. Stainless steel (SS) and copper material, the most common metallic mesh substrates, can be modified to become superhydrophobic adsorbents^15^. Hierarchical micro and nano roughness surfaces are fabricated by different methods such as acid erosion, colloidal assembly, rough polymer film, crystal growth, and chemical vapor deposition (CVD)^15,29–31^. Currently, alkylsilanes or perfluoroalkylsilanes, PDMS-based polymers, thiols, long alkyl chain fatty acids, perfluorinated polymers, and so on are used for decreasing surface energy. For example, chlorosilanes such as 1H, 1H, 2H, 2H-perfluoroctyldimethylchlorosilane (PFODMCS), dimethyldichlorosilane (DMDCS), and 1H, 1H, 2H, 2Hperfluorooctyltrichlorosilane (PFOTCS) can easily give surfaces the superhydrophobic property^14,32^.

Matin et al. reported the dip-coating of SS separator using different concentrations of silane materials, perfluo-rooctyltrichlorosilane^33^. They demonstrated that after seven cycles, water droplets on the superhydrophobic surface of the SS reached an angle of about 150.5°, and the resulting superhydrophobic adsorbent successfully separated the various types of oils with a 95% efficiency. Qiang et al. reported the fabrication of silane functionalized reduced Graphene oxide nanoribbons (GONR) coated polyurethane (PU) sponge composites via a facile dip-coating method. The produced porous superhydrophobic composite has good oil/water separation selectivity and capacity with high efficiency (> 97% after 10 cycles) and water contact angles of higher than 150°^34^. Khodaei et al. developed superhydrophobic aluminum with a nano/micro hierarchical surface structure by chemical etching and decorating nanoparticles with a silane-based nanocomposite coating (Al_2_O_3_ nanoparticles integrated TEOSGPTMS)^35^. Wang et al. demonstrated the easy production of superhydrophobic and superoleophobic cotton textiles modified with polysiloxane nanowires for oil/water separation. The low-surface-energy polysiloxane nanowires besides the hierarchical structure led to the cotton fabrics with great superhydrophobicity (WCAs 163°) and excellent stability^36^.

In this work, carbonyl iron particles were first reacted with Tetraethoxysilane, and then the surface was altered by FAS and HDMS in order to create superhydrophobic/superoleophilic and recyclable adsorbent particles. The characterization of the carbonyl iron modification particles with various silane groups was evaluated by using FT-IR, XRD, and VSM. The WCA study was then completed to quantitatively assess the particles' superhydrophobicity. The sorption capacity of superhydrophobic particles was also examined for oil/water separation and emulsion mixture under various circumstances.

## Experimental

### Materials

Absolute ethanol and Ammonia (25–28%wt.) were bought from DR-MOJALALI Medicines Co., Ltd, and Carbonyl Iron (CI) particles from BASF Company, Germany. Tetraethoxysilane (TEOS, 99.9%), Hexamethyldisilazane (HMDS, 98%) and 1H,1H,2H,2H-perfluorodecyltriethoxysilane (PFDTES or FAS, 97%) were bought from Sigma-Aldrich. All of the chemical reagents were utilized exactly as they were received, with no further purification.

### Synthesis

#### Synthesis of carbonyl iron with SiO_2_

First, 0.2 g of carbonyl iron was added into 40 mL of ethanol which contained 4 mL of ammonia solution. Subsequently, 0.2 mL of TEOS was added and sonicated in a sonication bath for 6 h. The solution was then three times vacuum-washed with ethanol in the following step. After that, it spent 24 h in an oven at 110 °C. Then, carbonyl iron particles were coated with SiO_2_(CI/SiO_2_).

#### Synthesis of carbonyl iron with SiO_2_-FAS

First, 60 mL of ethanol containing 6 mL of an ammonia solution was mixed with 0.2 g of CI/SiO_2_. After that, 0.4 mL of FAS was added, and it was sonicated for 8 h in a sonication bath. The solution was then three times vacuum-washed with ethanol in the following step. After that, it spent 24 h in an oven at 110 °C. After this period of time, carbonyl iron particles were given a coating of SiO_2_-FAS, denoted by the notation CI/SiO_2_@FAS.

#### Synthesis of carbonyl iron with SiO_2_-HMDS

First, 0.2 g of CI/SiO_2_ was mixed with 100 mL of ethanol and 8 mL of an ammonia solution. After that, 1 mL of HMDS was added, and the mixture was sonicated for 4 h in a sonication bath. The solution was then three times vacuum-washed with ethanol in the following step. After that, it spent 24 h in an oven at 110 °C. After this time, carbonyl iron particles were coated with SiO_2_-HMDS(CI/SiO_2_@HMDS).

### Characterization

Fourier transform infrared spectroscopy was used to examine the functional groups on the surface of carbonyl iron particles (FT-IR, THERMO, AVATAR). The crystal structure of carbonyl iron particles modified with different silane groups was investigated by analysis of the X-ray diffraction (XRD, Philips, PW1730). The magnetic properties of the modified particles were also measured using a vibrating sample magnetometer (VSM, Model No. 155, Magnet: Varian, V-7300). Furthermore, Jikan CAG-20, Jikan Co., and Image J^®^ 1.51i software investigated the oil and water contact angle of altered particles.

### Preparation of the oil-in-water emulsion and mixture

Oil and water were emulsified at 1% by weight in hexane. In this regard, to create a stable emulsion, one gram of oil was dissolved in 99 g of deionized water and swirled for 30 min. An ultrasonic probe (100 kW) was used for 10 min to minimize the size of oil droplets in water. It should be noted that stirring for longer periods caused the formation of nano-sized droplets. As a result, the oil–water emulsion was made by adding various oil concentrations to the water's surface, ranging from 0.1 to 1 mL. In addition, one mL of oil was added to the water's surface to create the oil–water mixture. The same method was used to make mixtures and emulsions of water with kerosene, silicon oil, and gasoline.

### Oil adsorption experiments

The efficacy and capability of modified materials were demonstrated using their sorption capacity. The following equation (Eq. (1)) can be used to determine the water or oil sorption capacity from the measurement of the weight of the liquid that is adsorbed due to placement or immersion:

1 $$ Q = (m_{e} - m_{0} )/m_{0} $$

While m_e_ and m_0_ are the material's weight before and after liquid sorption, respectively, and Q (g/g) is the amount of sorption capacity for various mixtures and emulsions, the oil separation's sorption capacity was estimated.

### Recycling test

Magnetic recycling of modified carbonyl iron particles has been investigated. Following oil adsorption, the superhydrophobic magnetic particles are collected, treated with chloroform and ethanol, and dried for 12 h at 60 °C. These particles will subsequently be employed in the oil sorption technique that follows. The reusability of hydrophobic magnetic particles was investigated over ten cycles.

## Results and discussion

### Characterization

**Figure 1:**
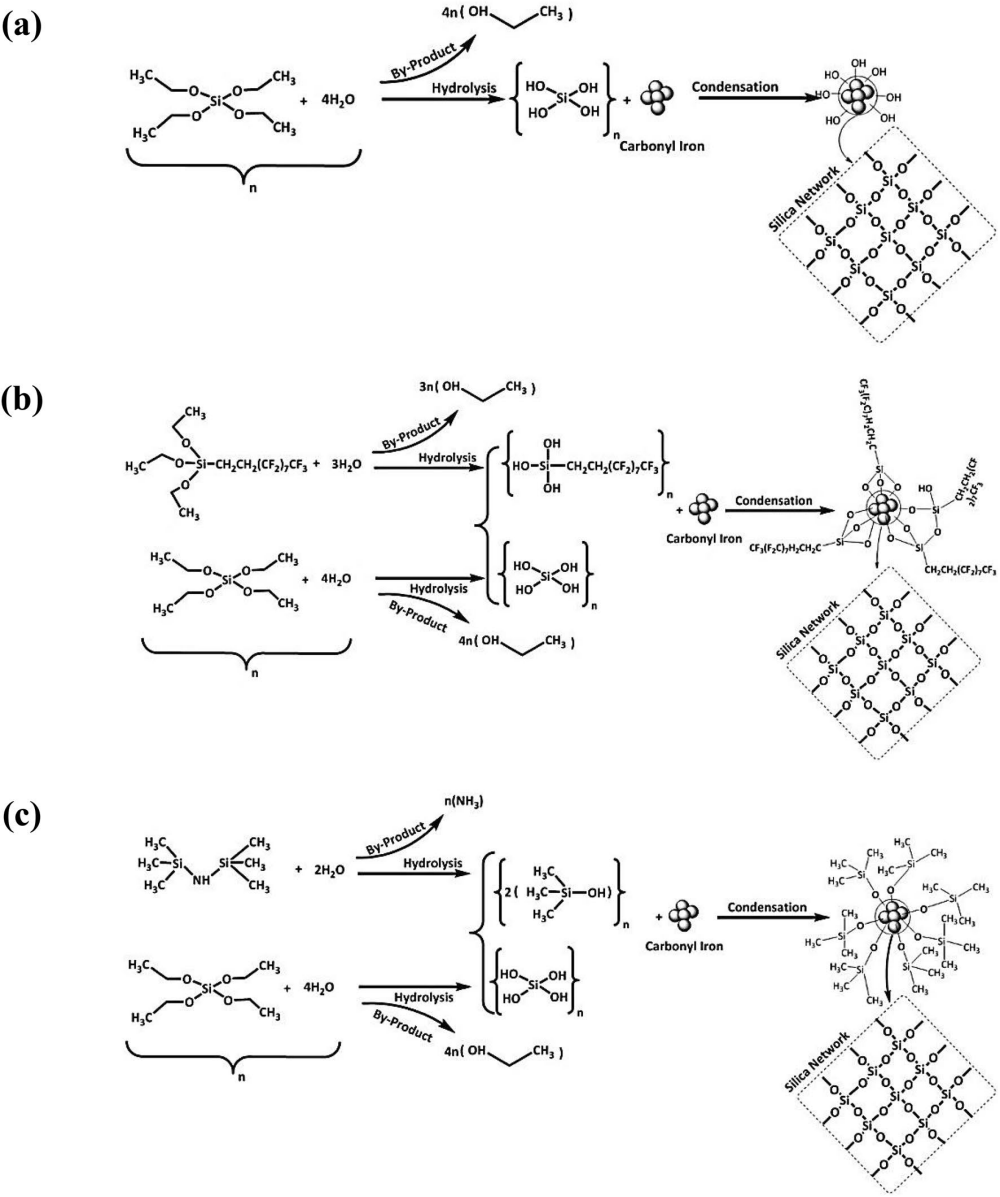
Mechanism of carbonyl iron reaction with (**a**) SiO_2_, (**b**) SiO_2_@FAS, and (**c**) SiO_2_@HDMS.

The reaction mechanism of different silane agents on the carbonyl iron particles has been shown in Fig. 1. The TEOS was hydrolyzed in the presence of ethanol and water, and Si–OR + H_2_O was converted to Si–OH + ROH. Then the silica network (Si–O–Si) was created through a condensation reaction on the surface of carbonyl iron particles according to Fig. 1a. FAS was then formed on the surface of carbonyl iron-SiO_2_ via a hydrolysis and condensation process (Fig. 1b). Similarly, as can be seen in Fig. 1c, the HDMS was hydrolyzed and condensed on the surface of carbonyl iron-SiO_2_. In addition, due to the sensitivity of conversion rates to the presence of acids and bases, ammonia solution was employed as a catalyst for all processes^1,2,37–40^.

**Figure 2:**
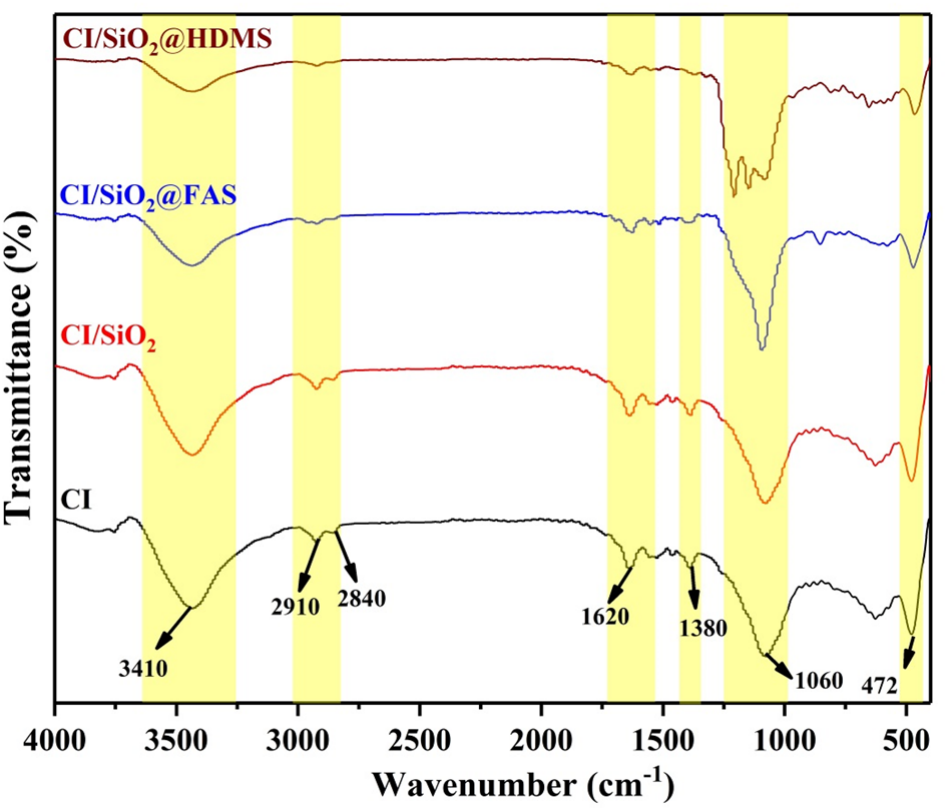
FTIR of carbonyl iron and modified hydrophobic particles with SiO_2_, SiO_2_@FAS, and SiO_2_@HDMS.

Functional groups have been examined using FT-IR analysis to confirm that iron carbonyl particles react with various silane groups. Figure 2 shows peaks related to different functional groups of SI, FAS, and HDMS on the surface of iron carbonyl particles. The spectra of CI particles modified with SiO_2_, FAS, and HDMS showed patterns typical of siloxane materials. The peaks of several silane groups, including Si–O–C, Si–O–Si, and Si–O–C, were assigned to a range of 1000 to 2000 cm^−1^^38,41^. On the FT-IR curve, the peak relative to Si–O–Fe was given 500 cm^−1^. Also, at 845 and 1255 cm^−1^, FT-IR signals linked with Si–C were found^37^. The wide band about 3500 cm^−1^ shows the stretching of several OH groups; this peak was gotten weaker by adding HDMS and FAS to the surface of CI/SiO_2_, indicating that the OH groups had been replaced by silane bands^40,42–44^.

**Figure 3:**
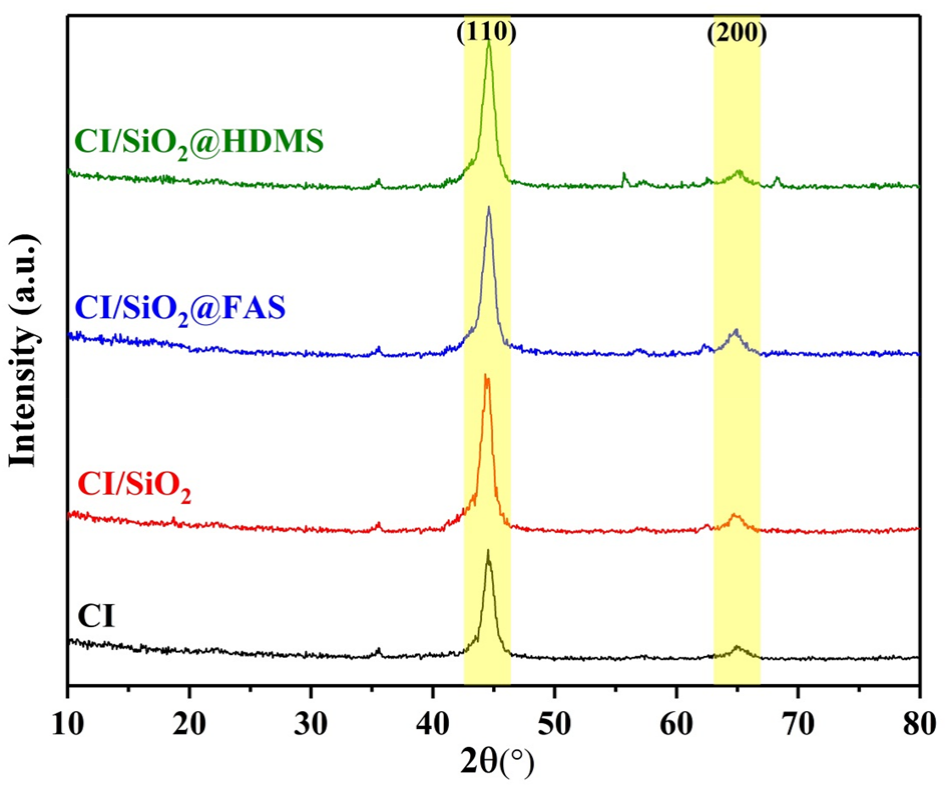
XRD of carbonyl iron and modified hydrophobic particles with SiO_2_, SiO_2_@FAS, and SiO_2_@HDMS.

XRD analysis was used to investigate oxidation or changes in the crystal structure that occur during synthesis. The XRD pattern for iron carbonyl particles modified with SiO_2_, SiO_2_@FAS, and SiO_2_@HDMS is shown in the Fig. 3. Then, the resulting XRD pattern revealed the 2θ of 44° given to (110), and 66° to (211) connected to the Fe peaks in the synthesized sample. No additional peak has been seen for any alterations other than these two peaks, proving that the oxidation reaction was not performed throughout the synthesis process^1^.

**Figure 4:**
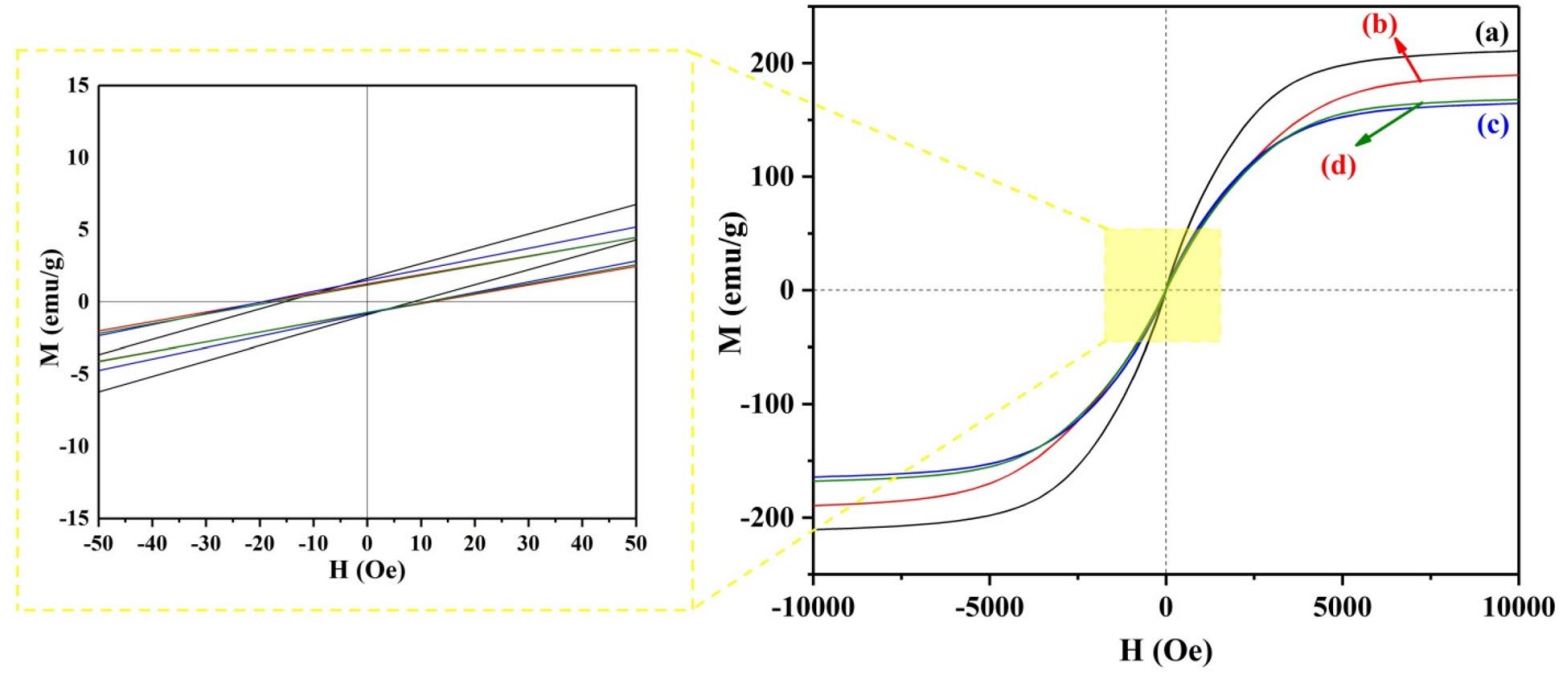
VSM curve for modified hydrophobic particles (**a**) bare CI, (**b**) SiO_2_, (**c**) SiO_2_@FAS, and (**d**) SiO_2_@HDMS.

The magnetic characteristics of carbonyl iron particles with various silane coupling agents were studied using a VSM and the results are depicted in Fig. 4. In the 10 kOe field, it was shown that carbonyl particles had strong magnetic permeability with magnetic saturation of 210, 188, 167, and 166 emu/g for bare CI, CI/SiO_2_, CI/SiO_2_@FAS, and CI/SiO_2_@HDMS, respectively. The modified particles still exhibit high saturation magnetism despite having a non-magnetic covering. In addition, the magnetization curve shows only a small amount of magnetic hysteresis as well as coercivity (H_c_) and remnant magnetism (M_r_) for modified carbonyl iron particles. Other magnetic properties of samples, H_c_ and M_r_, are summarized in Table 1.

**Table 1:** Summary of magnetic properties of bare CI, SiO_2_, SiO_2_@FAS, and SiO_2_@HDMS.

Sample	H_c_ (Oe)	M_r_ (emu/g)
CI	8.57	1.63
CI/SiO_2_	11.84	1.25
CI/SiO_2_@FAS	10.96	1.48
CI/SiO_2_@HDMS	11.15	1.17

### Wettability properties

One of the most crucial factors to consider when assessing a material's wettability characteristics is the water contact angle (WCA) or oil contact angle (OCA). Hence, the sessile water droplet method with a volume of 5 µL was utilized so that the wettability of particles (CI, CI/SiO_2_, CI/SiO_2_@FAS, and CI/SiO_2_@HMDS) could be evaluated. The powder particles were compacted between two glass substrates to form a level layer. Utilizing a Hamilton microliter syringe, vertical liquid droplets were put on the accumulated particles. Five contact angle measurements were carried out in various places, and the findings were provided according to their mean value. Image J^®^ 1.51i software was used to process all liquid droplet photographs^45^.

**Figure 5:**
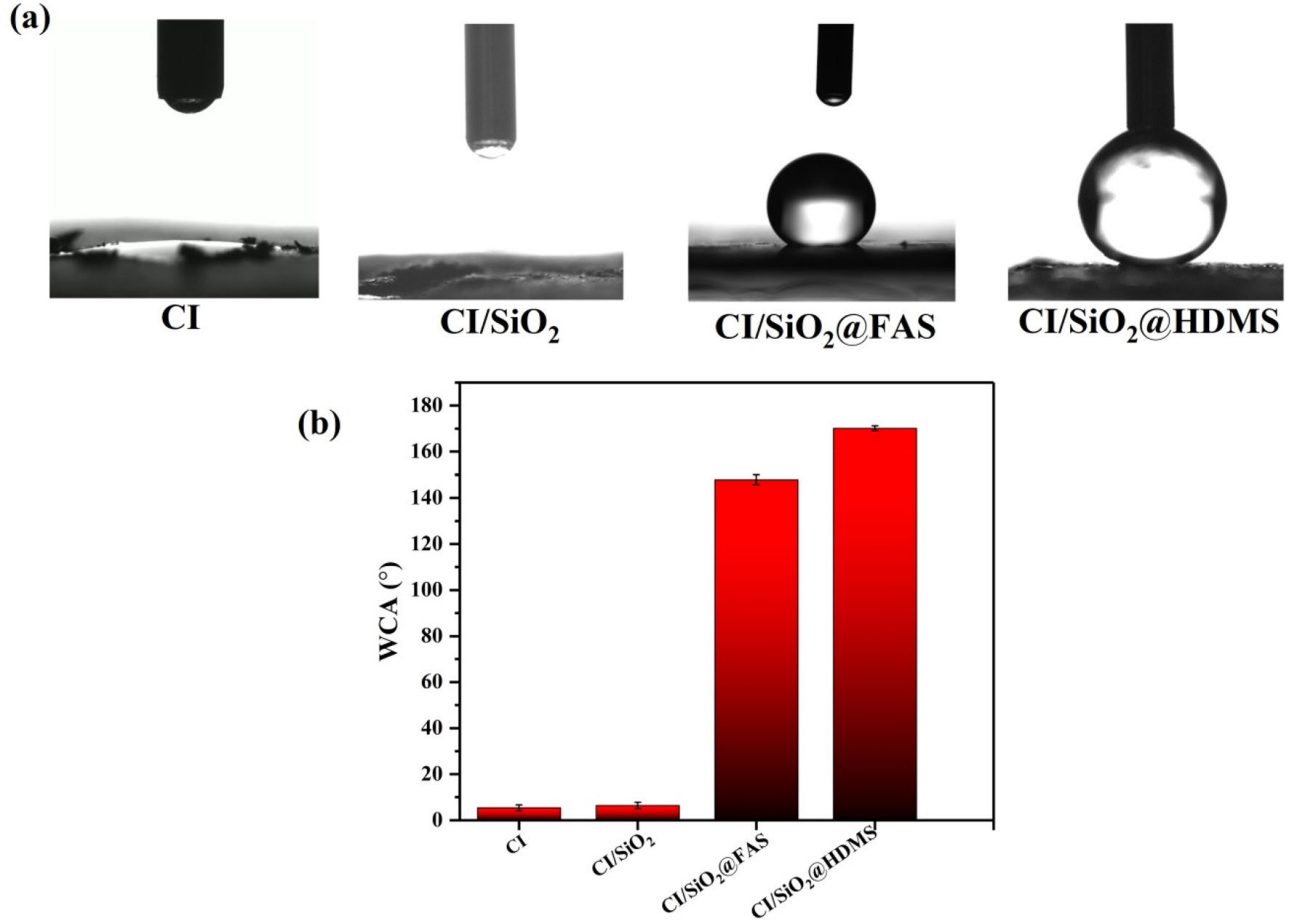
(**a**) The digital photos and (**b**) WCA variation of water droplets on the layered particles.

Figure 5 depicts the digital photographs and the WCA variation of the liquid droplets on the stacked particles. According to the findings, the WCA values for CI, CI/SiO_2_, CI/SiO_2_@FAS, and CI/SiO_2_@HMDS were 5.4° ± 1.3°, 6.4° ± 1.4°, 151.9° ± 2.1°, and 170.1° ± 1.1°, respectively. In addition, it is essential to point out that the OCAs of a variety of oils were found to be equivalent to 0°. The findings demonstrate that both CI and CI/SiO_2_ possess superhydrophilicity and superoleophilicity. Because of this, the use of these materials in processes that separate oil and water cannot be considered a viable option. On the other hand, CI/SiO_2_@FAS and CI/SiO_2_@HMDS exhibited features of superhydrophobicity and superoleophilicity properties. The production of a high water-repellent substrate is specifically dependent on two primary factors: high surface roughness and the use of material with low surface energy. The different processes of hydrolysis and condensation that take place on the sample's surface lead the materials to behave differently in terms of their propensity to adsorb water^46,47^.

### Sorption process

Due to their unique superhydrophobicity/superoleophilicity qualities, CI/SiO_2_@FAS and CI/SiO_2_@HMDS can be considered very effective adsorbents in oil/water mixtures and emulsion separations. In addition, the ferromagnetic responses of particles can contribute to the collection and reuse processes. For the separation of oils from the oil/water mixture in the batch system, the as-prepared modified particles were used. Initially, 300 mg of red-dyed oil was sprinkled onto the water's surface. Subsequently, the magnetic particles were left on the oil spill for 5 min, and time was given. The patch of slick oil began to reduce at that point.

**Figure 6:**
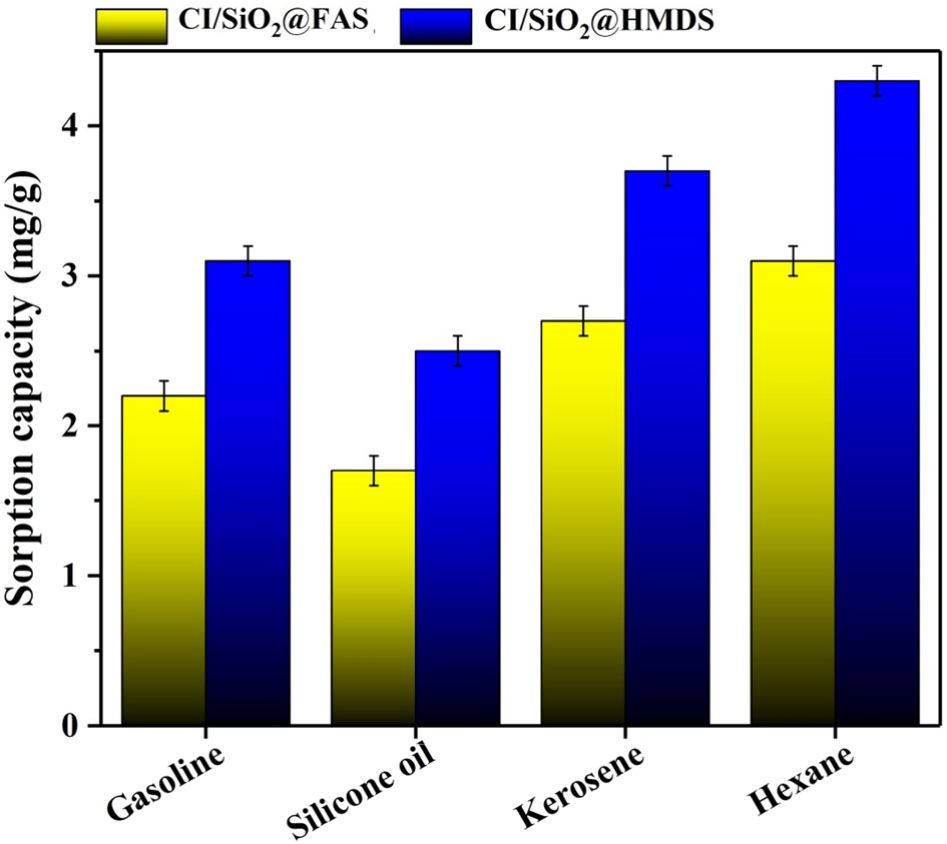
The sorption capacity of CI/SiO_2_@FAS, and CI/SiO_2_@HMDS.

Over time, it was discovered that superhydrophobic/superoleophilic powders intuitively capture and cover oil droplets before sinking into the water. After collecting the oil-particle combination with a magnet, the ultimate mass of the marble was calculated. The measured sorption capacity for oils, including hexane, kerosene, silicone oil, and gasoline, were in the ranges of 1.7–3.1 g/g and 2.5–4.3 g/g for CI/SiO_2_@FAS, and CI/SiO_2_@HMDS, respectively (Fig. 6), which can compete with other used absorbents (Table 2). The results showed that the highest and lowest sorption capacity belonged to hexane and silicone oil, respectively.

**Table 2:** Sorption capacity of various particles.

Particle	Grafting agent	Water contact angle (°)	Sorption capacity (g/g)	Ref
Magnetic hollow carbon microspheres	Oleic acid	150	6.5–10.8	^48^
Spiky nickel nanowires	Octadecyltrichlorosilane	169.17	3.86–5.27	^4^
Covalent organic frameworks	Fluorine-containing groups	151	0.45–2.25	^49^
Carbonyl iron @glucose	Stearic acid	169	2.5–4.1	^1,2^
Core–Shell Fe_2_O_3_@C	Polysiloxane	162.9	3.7	^50^
Fe_3_O_4_@C/Cu	–	138.4	3.05–3.38	^51^
S-MIL-101(Cr)	Octadecylamine	156	1.18–2.81	^52^
ZIF-8	Polydimethylsiloxane	142	0.7–2.5	^53^
Iron papilla-like particles	Lauric acid	164.5	2	^54^
ZnO	Trimethoxy(octadecyl)silane	160	1.5	^55^
CaCO_3_	Oleic acid	155	0.99	^56^
Flower-like ZnO microparticles	Octadecyl trimethoxysilane	154	0.5	^57^
Iron particles	1-dodecanethiol	164	0.28–0.45	^58^
Carbonyl iron/SiO_2_	1H,1H,2H,2H-perfluorodecyltriethoxysilane (FAS)	151.9	1.7–3.1	Present study
Carbonyl iron/SiO_2_	Hexamethyldisilazane (HDMS)	170.1	2.5–4.3	Present study

**Figure 7:**
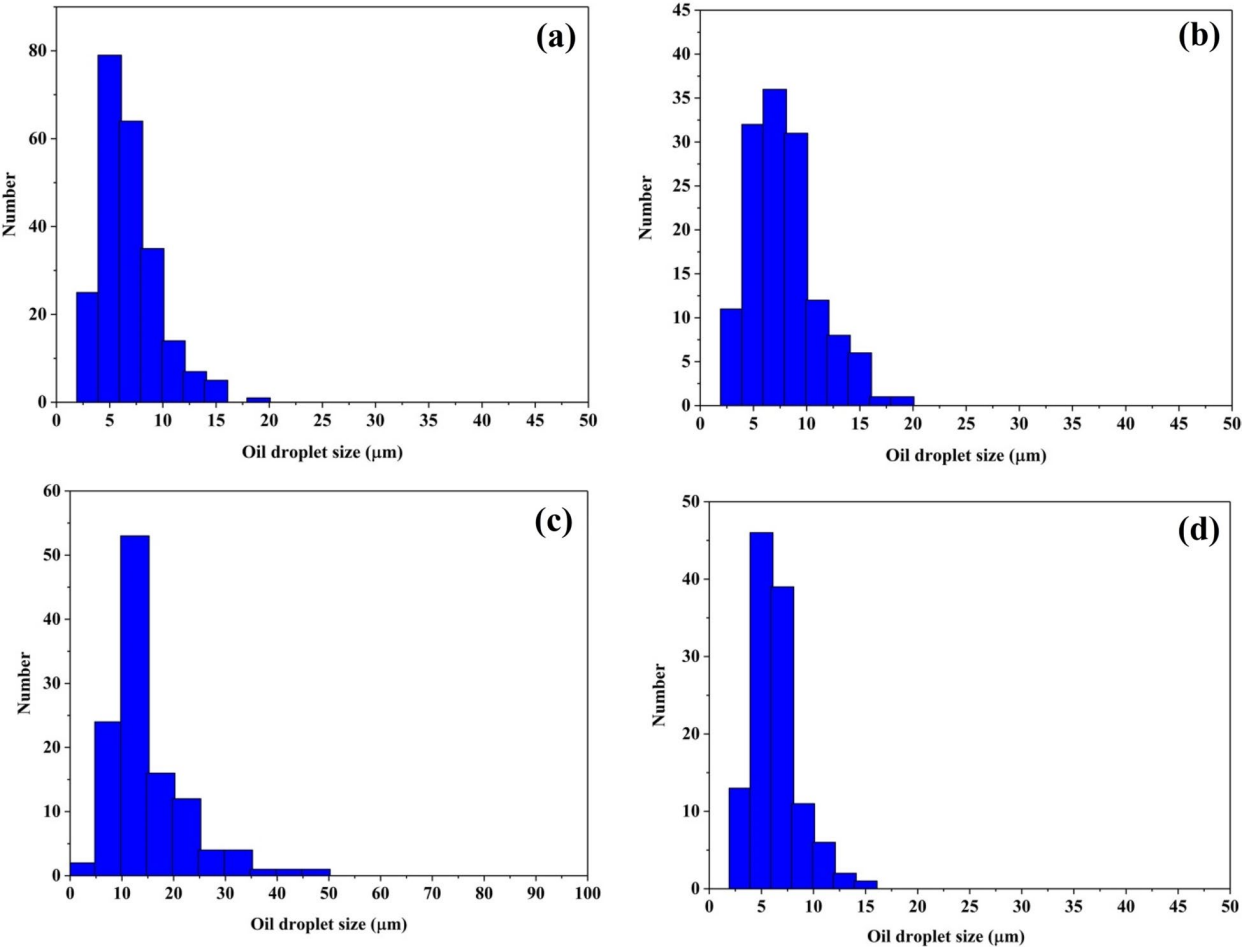
Oil droplet size distributions of various surfactant-free oil/water emulsions; (**a**) hexane, (**b**) silicone oil, (**c**) gasoline, and (**d**) kerosene.

**Figure 8:**
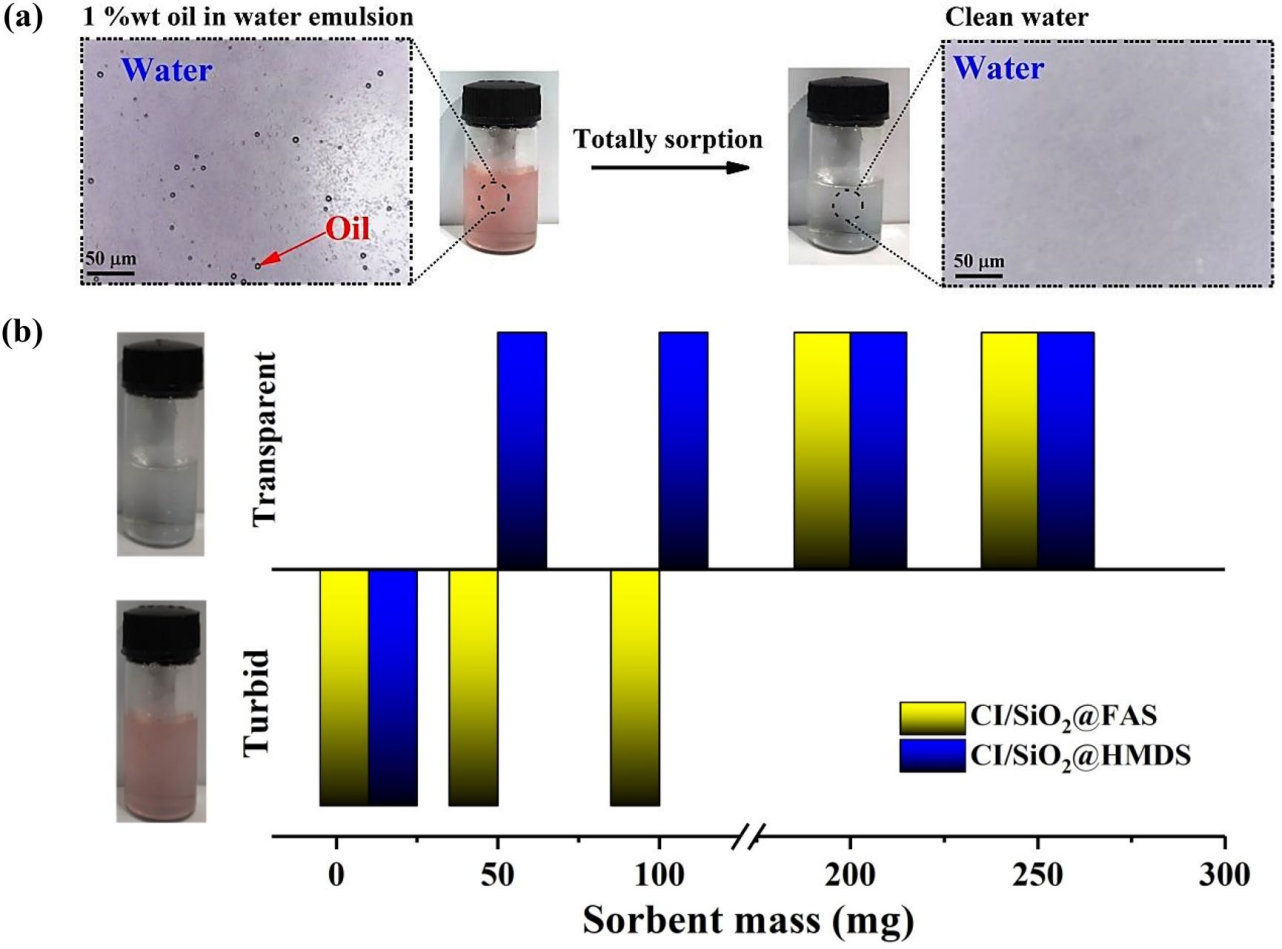
(**a**) Schematic illustration of separation of tiny oil-droplets from the oil-in-water emulsion, (**b**) the effect of sorbent mass on the turbidity or transparency of final filtrate solution from the hexane-in-water emulsion.

In the recent decade, numerous studies have been done to address oil/water emulsion separation. Foams, metal meshes, and fabric materials are some technologies that can be used to separate oil from oil emulsions in water; however, these approaches all have their drawbacks^59–62^. In this context, using particles that are both superhydrophobic and superoleophilic can be an effective strategy for separating oil and water emulsions. In the current investigation, a number of 1%w/w oil/water emulsions were created. These emulsions included hexane, kerosene, silicone oil, and gasoline. The size distributions of oil droplets in different surfactant-free oil/water emulsions were shown in Fig. 7. Hexane, kerosene, silicone oil, and gasoline all had an average droplet size of 6.54 ± 1.78, 8.32 ± 1.54, 13.49 ± 6.94, and 6.12 ± 2.40 µm, respectively, when they were homogeneously distributed in the water. It is important to highlight that the images obtained from optical microscopy demonstrated that the emulsions kept their stability over one day. Particles ranging in mass from 5 to 250 mg were added directly to 3 mL of emulsions and vigorously vortexed for 10 min. The upper aqueous solution was removed after the oily particles were separated from the water using a magnetic field to evaluate the water profile. A schematic illustration of the separation of tiny oil droplets from the oil-in-water emulsion is illustrated in Fig. 8a. Initially, a low dosage of superhydrophobic/superoleophilic particles cannot separate oil droplets totally and the final solution looks turbid. By increasing the mass of sorbent in the system, the final solution becomes transparent, which is different for each sorbent and oil (Fig. 8b). For instance, by adding 50 mg of CI/SiO_2_@FAS particles to 1%w/w hexane/water emulsion, the resultant final filtrate solution was semitransparent. In contrast, by adding the same mass of CI/SiO_2_@HDMS, the final solution can become transparent. The lowest mass of particles to separate a 1%w/w hexane/water emulsion was 50 and 200 mg for CI/SiO_2_@HDMS and CI/SiO_2_@HDMS, respectively. As a result, the powdery materials such as CI/SiO_2_@HDMS and CI/SiO_2_@HDMS particles have the ability to contact directly with emulsion droplets, which can be suggested as very effective systems in separating oil droplets from oil-in-water emulsions.

### Reusability and chemical durability of the superhydrophobic sample

**Figure 9:**
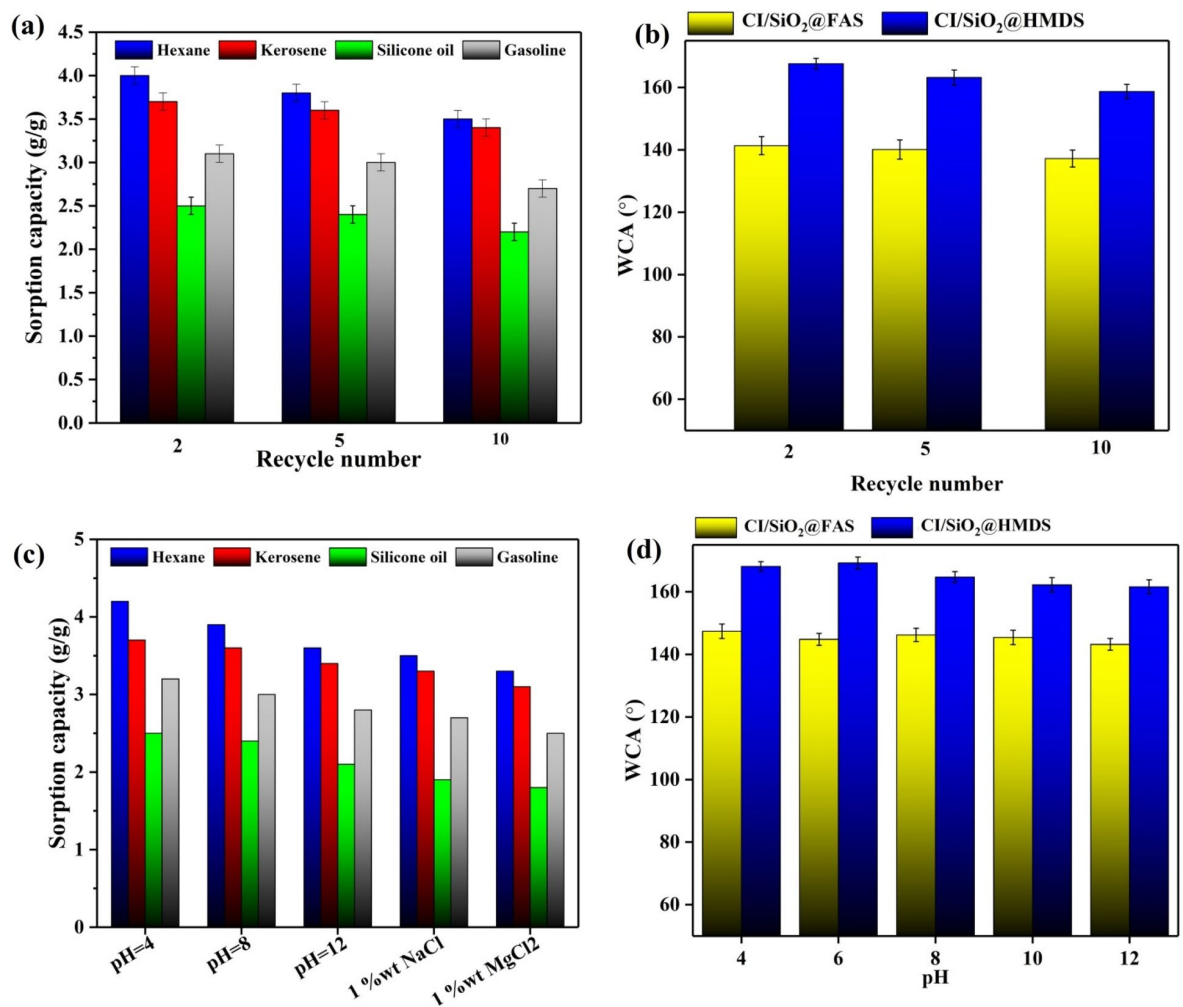
(**a**) Recyclability of the CI/SiO_2_@HDMS in different regeneration cycles, (**b**) water contact angle variations in different cycles, (**c**) the performance of CI/SiO_2_@HDMS for sorption oils under various circumstances, and (**d**) water contact angle variations for immersion at different pH values for one week.

The sorption capacities of recyclable superhydrophobic/superoleophilic CI/SiO_2_@HDMS particles for oils are presented in Fig. 9a, which indicates that the sorption capacity has not significantly changed even after 10 separation cycles. For instance, the sorption capacity of hexane revealed that after ten cycles, the original sorption capacity was reduced by just 0.8 g/g (4.3 to 3.5 g/g), which is a relatively small amount. The shift in WCAs is responsible for the fluctuating sorption capacities, as this has been demonstrated. Figure 9b displays a slight decreasing trend in the water contact angles (WCA) of the droplets on the regenerated particles, which is consistent with the sorption capacity data. As shown in Fig. 9c, the as-prepared particles can meet practical needs in severe and hard settings since their sorption capacity for oils remains roughly constant even in acidic, alkaline, and high-saline environments. Wetting behavior and chemical stability under a wide range of circumstances is the main problem with superhydrophobic/superoleophilic surfaces (acidic, alkaline, and saline solution). WCA variations were also used to examine the effects of varying the pH of the prepared superhydrophobic/superoleophilic particles for a week. Good hydrophobicity is indicated by WCA values of more than 145°, as seen in Fig. 9d. Superhydrophobic samples are shown to have outstanding chemical durability and physical stability in steady or flowing settings, making them a prime candidate for oil/water separation technology.

## Conclusion

In conclusion, the superparamagnetic superhydrophobic/superoleophilic, as well as reusable and durable carbonyl iron/SiO_2_ particles were prepared through a simple grafting route. Silane coupling agents, namely 1H,1H,2H,2H-perfluorodecyltriethoxysilane (FAS) and Hexamethyldisilazane (HDMS), were used to illustrate the effect of modifying surface wettability of the resulted samples. The reaction mechanism of different silane agents on the carbonyl iron particles has shown that FAS and HDMS were formed on the surface of carbonyl iron-SiO_2_ via a hydrolysis and condensation process via the creation of a silica network. CI/SiO_2_@FAS and CI/SiO_2_@HMDS exhibited ferromagnetic properties with magnetic saturation of 167 and 166 emu/g for CI/SiO_2_@FAS and CI/SiO_2_@HDMS, respectively. Also, the measured sorption capacities of CI/SiO_2_@FAS and CI/SiO_2_@HMDS were in the ranges of 1.7–3.1 g/g and 2.5–4.3 g/g for different kinds of oils, which can compete with other used absorbents. Also, the sorption capacities of recyclable superhydrophobic/superoleophilic particles indicated good recyclability and reusability even after ten separation cycles. Besides, superhydrophobic samples have outstanding chemical durability and physical stability in acidic, alkaline, and high-saline circumstances, making them a prime candidate for oil/water separation technology. Consequently, CI/SiO_2_@HDMS and CI/SiO_2_@HDMS particles have the ability to contact directly with emulsion droplets, which can be suggested as very effective systems in separating oil droplets from oil-in-water emulsions.

## Data Availability

All data generated or analyzed data for the experimental part of this study are included in this published article. The data that support the findings of this study are available from the corresponding author, [Yahya Rabbani], upon reasonable request. Moreover, all other data that support the plots within this paper and other findings of this study are available from the corresponding author upon reasonable request.
